# Supplementary material for: Father’s adolescent body silhouette is associated with offspring asthma, lung function and BMI through DNA methylation
Source: Commun Biol. 2025 May 24;8:796. doi: 10.1038/s42003-025-08121-9 (PMC12102279; doi:10.1038/s42003-025-08121-9)
Supplement: Supplementary file 1 — Supplementary Information [file 42003_2025_8121_MOESM1_ESM.pdf]

Father's adolescent body silhouette is associated with offspring asthma, lung function and BMI through DNA methylation

Negusse Tadesse Kitaba[1], Toril Mørkve Østergaard[2], Marianne Lønnebotn[3], Simone Accordini[4], Francisco Gómez Real[5], Andrei Malinovschi[6], Anna Oudin[7], Bryndis Benediktsdottir[8], Francisco Javier Callejas González[9], Leopoldo Palacios Gómez[10], Mathias Holm[11], Nils Oskar Jõgi[6], Shyamali C. Dharmage[12], Svein Magne Skulstad[2], Vivi Schlünssen[13], Cecilie Svanes[2, 14]*#, John W. Holloway[1,15]#

Supplementary data (https://doi.org/10.5258/SOTON/D3067)
Note EWAS list and label used on each excel sheet.

1. Summary statistics for association father body silhouette and offspring DNA methylation at FDR <=0.05.
    a. Data 1A EWAS dmCpGs.xlsx
    b. Data 1B EWAS dmCpGs Mvalue and beta compared_VB.xlsx and
2. Differential methylated region Data 2 DMR.xlsx
3. Look up of dmCpGs overlap with transcription factor binding sites from eForege-TF q-value<0.05. Data 3 TF.xlsx
4. Look-up of dmCpGs in EWAS Atlas effect methylation on gene expression(meQTL) p<0.05. Data 4 eQTL.xlsx
5. Pathway signalling enrichment from methylR. Data 5 methylRR.xlsx
6. Gene ontology terms, gene description and lipid traits from String database for dmCpGs at FDR<=0.05. Data 6 Gene ontology terms.xlsx
7. Pathway signalling enrichment from KEGG and Wikipathway. Data 7 KEGG Wikipathway.xls
8. Lookup overlap GWAS catalogue FUMAGWAS and enrichr dbGAP. Data 8 Overlab GWAS Catalague.xlsx
9. Look-up of dmCpGs in EWAS Atlas for overlap of known traits p<0.05 Data 9 Overlap EWASatlas.xlsx
10. Lookup SNP methylation interaction for meQTL from goDMC and meQTL EPIC database at p > 0.05. Data 10A meQTL goDMC.xlsx and Data 10B meQTL EPIC Database.xlsx
11. Lookup imprintome: Data 11 imprinted gene and metastable epialleles.xlsx
12. Association Offspring health outcome.
    a. Data 12A BMI.xlsx
    b. Data 12B Asthma.xlsx
    c. Data 12C Lung function.xlsx
13. Association Offspring BMI and body silhouette: Data 13 offspring BMI and body silhouette.xlsx
14. Lookup of overlap of obesity related gene : Data 14 Obesity related dmCpG Open Targets and DisGeNET.xlsx

Supplementary Figures

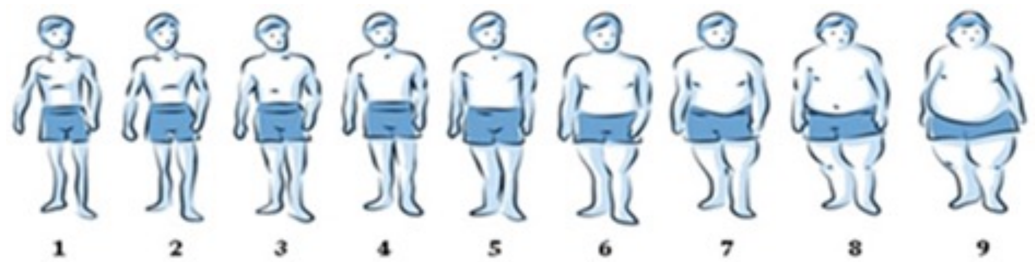

Supplemental Fig. 1 Body silhouettes in scale 1: 9.

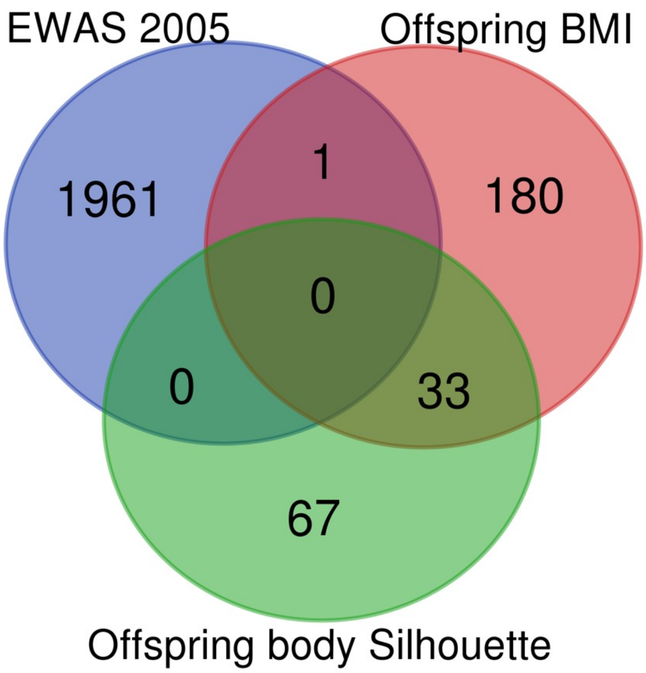

Supplemental Fig. 2 Shared dmCpGs between EWAS of offspring current BMI (red), EWAS of offspring current body silhouette (green) and 1962 unique dmCpGs associated with father body silhouette.

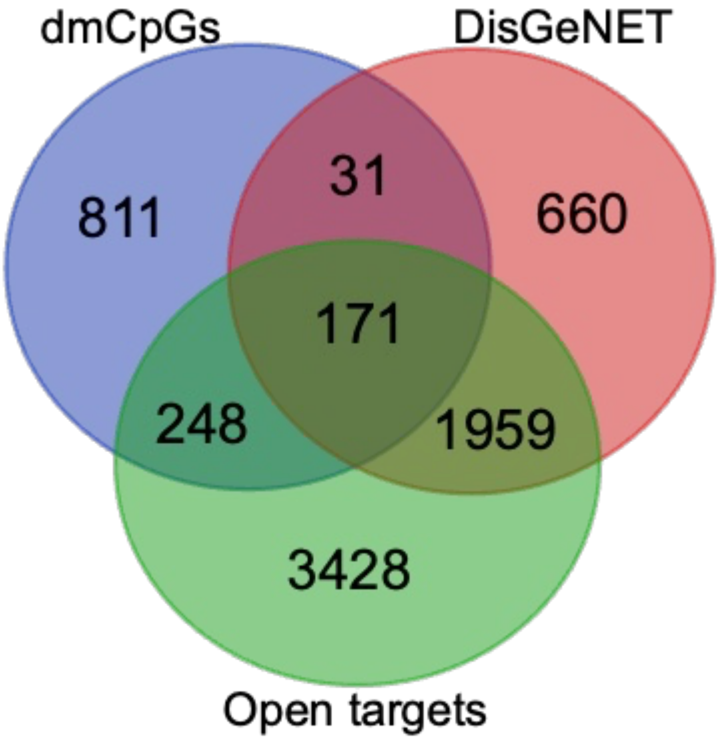

Supplemental Fig. 3: Shared obesity-related genes between RHINESSA EWAS genes (dmCpGs), Open target and DisGeNET database.
