# Supplementary material for: Father’s adolescent body silhouette is associated with offspring asthma, lung function and BMI through DNA methylation
Source: Commun Biol. 2025 May 24;8:796. doi: 10.1038/s42003-025-08121-9 (PMC12102279; doi:10.1038/s42003-025-08121-9)
Supplement: Supplementary file 2 — Reporting summary [file 42003_2025_8121_MOESM2_ESM.pdf]

nature portfolio

Corresponding author(s): Prof Cecilie Svanes
Address: Centre for International Health, Department of Global Public Health and Primary Care, University of Bergen, Norway, Årstadveien 21, 5009 Bergen, Norway
Tel: +47 908 92762 or +47 55588372
Email: cecilie.svanes@helse-bergen.no

Last updated by author(s): Apr 1, 2025

Reporting Summary

Nature Portfolio wishes to improve the reproducibility of the work that we publish. This form provides structure for consistency and transparency in reporting. For further information on Nature Portfolio policies, see our Editorial Policies and the Editorial Policy Checklist.

Statistics

For all statistical analyses, confirm that the following items are present in the figure legend, table legend, main text, or Methods section.

n/a	Confirmed	
☐	☒	The exact sample size (*n*) for each experimental group/condition, given as a discrete number and unit of measurement
☐	☒	A statement on whether measurements were taken from distinct samples or whether the same sample was measured repeatedly
☐	☒	The statistical test(s) used AND whether they are one- or two-sided<br>*Only common tests should be described solely by name; describe more complex techniques in the Methods section.*
☐	☒	A description of all covariates tested
☐	☒	A description of any assumptions or corrections, such as tests of normality and adjustment for multiple comparisons
☐	☒	A full description of the statistical parameters including central tendency (e.g. means) or other basic estimates (e.g. regression coefficient) AND variation (e.g. standard deviation) or associated estimates of uncertainty (e.g. confidence intervals)
☐	☒	For null hypothesis testing, the test statistic (e.g. *F*, *t*, *r*) with confidence intervals, effect sizes, degrees of freedom and *P* value noted<br>*Give P values as exact values whenever suitable.*
☒	☐	For Bayesian analysis, information on the choice of priors and Markov chain Monte Carlo settings
☒	☐	For hierarchical and complex designs, identification of the appropriate level for tests and full reporting of outcomes
☐	☒	Estimates of effect sizes (e.g. Cohen's *d*, Pearson's *r*), indicating how they were calculated

Our web collection on statistics for biologists contains articles on many of the points above.

Software and code

Policy information about availability of computer code

Data collection	No new code generated, all code used for this analysis can be provided on request. The custom code used to generate graphics are available at GitHub repository: https://github.com/negusse2025/EWAS-of-Father-s-adolescent-body-silhouette-.git.
Data analysis	R packages as detailed in manuscript.

For manuscripts utilizing custom algorithms or software that are central to the research but not yet described in published literature, software must be made available to editors and reviewers. We strongly encourage code deposition in a community repository (e.g. GitHub). See the Nature Portfolio guidelines for submitting code & software for further information.

Data

Policy information about availability of data

All manuscripts must include a data availability statement. This statement should provide the following information, where applicable:

- Accession codes, unique identifiers, or web links for publicly available datasets
- A description of any restrictions on data availability
- For clinical datasets or third party data, please ensure that the statement adheres to our policy

Provide your data availability statement here.

Research involving human participants, their data, or biological material

Policy information about studies with human participants or human data. See also policy information about sex, gender (identity/presentation), and sexual orientation and race, ethnicity and racism.

Reporting on sex and gender	We have used sex throughout the manuscript as all methylation data was checked for agreement with reported sex as part of the Qc steps.<br><br>Sex stratified analyses are reported in addition to combined offspring groups.
Reporting on race, ethnicity, or other socially relevant groupings	White European ethnicity is used as a term to describe the study population. This was self-reported by participants and not based on genetic ancestry. .
Population characteristics	In this study, 339 offspring-father pairs with complete data on fathers' body silhouettes and offspring DNA methylation were included. These participants were from six study centres (Aarhus, Denmark; Albacete/Huelva, Spain; Bergen, Norway; Melbourne, Australia; Tartu, Estonia). Offspring had a median age of 26 years. Outcome measures included BMI, diagnosis of asthma, lung function.
Recruitment	Parental data was retrieved from the population-based European Community Respiratory Health Survey (ECRHS, www.ecrhs.org) and/or the Respiratory Health in Northern Europe (RHINE, www.rhine.nu) studies. Offspring were identified using population registries and parental report.
Ethics oversight	Sweden (multicenter: Uppsala, Umeå and Göteborg) screening Regional Ethical Review Board in Uppsala Dnr 2013/ 352 Sweden (multicenter: Uppsala, Umeå and Göteborg) clinical Regional Ethical Review Board in Uppsala Dnr 2016/ 023 Melbourne, Australia Alfred Hospital Human Research Ethics Committee HREC/ 17/Alfred/ 144 Iceland, Reykjavik questionnaire The National Bioethics Committee VSN-13-190 Iceland, Reykjavik clinical The National Bioethics Committee VSN-16-070 Denmark Ethical Scientific Committee for Mid Region Jylland, Denmark 1-10-72-301-15 Spain, Huelva Comité de ética de la investigación de la provincia de Huelva(Research Ethics Committee of the Province of Huelva) Approval date April 4, 2013 Spain, Albacete Comité ético de investigación clínica del Complejo Hospitalatio Universitario de Albacete. (Ethic Committee of clinical Research of the University Hospital Complex of Albacete) Institutional Review Board IRB 00006998 Estonia, Tartu Research Ethics Committee of the University of Tartu (UT REC) 233/ T-7 Norway, Bergen Regional Committee of Medical and Health Research Ethics, Rec West 2012/1077

Note that full information on the approval of the study protocol must also be provided in the manuscript.

Field-specific reporting

Please select the one below that is the best fit for your research. If you are not sure, read the appropriate sections before making your selection.

☒ Life sciences ☐ Behavioural & social sciences ☐ Ecological, evolutionary & environmental sciences

For a reference copy of the document with all sections, see nature.com/documents/nr-reporting-summary-flat.pdf

Life sciences study design

All studies must disclose on these points even when the disclosure is negative.

Sample size	Pragmatic sample size ustilising all available offspring-father pairs with complete data on fathers' body silhouettes and offspring DNA methylation
Data exclusions	NA
Replication	No replication cohort with equivalent data on parental an doffspring generations has been identifed.
Randomization	NA

Blinding	NA

Reporting for specific materials, systems and methods

We require information from authors about some types of materials, experimental systems and methods used in many studies. Here, indicate whether each material, system or method listed is relevant to your study. If you are not sure if a list item applies to your research, read the appropriate section before selecting a response.

Materials & experimental systems

n/a	Involved in the study
☒	☐ Antibodies
☒	☐ Eukaryotic cell lines
☒	☐ Palaeontology and archaeology
☒	☐ Animals and other organisms
☐	☒ Clinical data
☒	☐ Dual use research of concern
☒	☐ Plants

Methods

n/a	Involved in the study
☒	☐ ChIP-seq
☒	☐ Flow cytometry
☒	☐ MRI-based neuroimaging

Clinical data

Policy information about clinical studies

All manuscripts should comply with the ICMJE guidelines for publication of clinical research and a completed CONSORT checklist must be included with all submissions.

Clinical trial registration	NA - not an interventional trial
Study protocol	NA - not an interventional trial
Data collection	Description of data collection available in: Svanes C, Johannessen A, Bertelsen RJ, Dharmage S, Benediktsdottir B, Bråbäck L, Gislason T, Holm M, Jõgi O, Lodge CJ, Malinovschi A, Martinez-Moratalla J, Oudin A, Sánchez-Ramos JL, Timm S, Janson C, Real FG, Schlünssen V; RHINESSA International Collaboration. Cohort profile: the multigeneration Respiratory Health in Northern Europe, Spain and Australia (RHINESSA) cohort. BMJ Open. 2022 Jun 2;12(6):e059434. doi: 10.1136/bmjopen-2021-059434. PMID: 35654464; PMCID: PMC9163543.
Outcomes	NA - not an interventional trial

Plants

Seed stocks	NA
Novel plant genotypes	NA
Authentication	NA
